# First detection and transatlantic introduction of Influenza A(H3N2) subclade K (J.2.4.1) into Ecuador: insights from genomic sentinel surveillance

**DOI:** 10.3389/fpubh.2026.1839338

**Published:** 2026-07-31

**Authors:** Alfredo Bruno, Domenica de Mora, Maritza Olmedo, Jimmy Garcés, Denisses Portugal, Lourdes Pardo, Miguel Angel Garcia-Bereguiain

**Affiliations:** 1 Instituto Nacional de Salud Pública e Investigación, Guayaquil, Ecuador; 2 Universidad Agraria del Ecuador, Guayaquil, Ecuador; 3 Instituto Nacional de Salud Pública e Investigación, Cuenca, Ecuador; 4 One Health Research Group, Universidad de Las Américas, Quito, Ecuador

**Keywords:** Ecuador, epidemiology, genomic surveillance, Influenza A(H3N2), subclade K (J.2.4.1)

## Abstract

**Background:**

The rapid evolution of Influenza A(H3N2) requires constant global monitoring. This study analyzes the genomic characteristics and the first introduction of the emerging subclade K (J.2.4.1) in Ecuador, utilizing publicly available data to trace its intercontinental movement.

**Methods:**

The hemagglutinin (HA) protein of an Influenza A(H3N2) strain isolated from an Ecuadorian patient was analyzed. A Bayesian phylogenetic reconstruction was performed using BEAST v1.10.4 with 251 global sequences. A molecular clock was applied to estimate the time of most recent common ancestor (TMRCA). Substitutions were screened using the FluSurver tool.

**Results:**

Phylogenetic analysis clustered the Ecuadorian isolate within a monophyletic group of European origin (Northern Spain). The estimated introduction occurred in late November 2025, aligning with epidemiological links to Ecuadorian migrants travelling from Spain for Christmas holidays. Molecular screening identified HA1 substitutions annotated in FluSurver as mutations of interest, including S160N, N174D, I176K, Q189R, and Y211F. S160N was associated with the creation of a potential N-glycosylation site. The NA protein harbored the N358D substitution, which was annotated in FluSurver as having a reported association with neuraminidase inhibitor susceptibility.

**Conclusion:**

The introduction of subclade K into South America illustrates the high efficiency of transatlantic travel as a conduit for viral dispersal. This study supports the use of open-access genomic surveillance as a critical sentinel tool for global health security.

## Introduction

The evolutionary trajectory of Influenza A(H3N2) is characterized by rapid antigenic drift, frequent clade turnover, and recurrent changes in the hemagglutinin (HA) glycoprotein, all of which necessitate continuous genomic surveillance to inform vaccine strain selection and detect emerging variants. Previous studies have shown that the evolutionary success of A(H3N2) is largely driven by substitutions in HA that can alter antibody recognition, often accompanied by changes in glycosylation patterns and rapid replacement of circulating clades. In addition, changes in neuraminidase (NA) may contribute to antigenic evolution and antiviral susceptibility profiles. These features make A(H3N2) particularly prone to recurrent seasonal epidemics and reinforce the need for genomic surveillance to identify emerging clades with potential epidemiological relevance (1–4).

In late 2025, a new variant, subclade K (J.2.4.1), emerged within the 2a.3a.1 clade and was associated with early and intense influenza activity in parts of the Northern Hemisphere, particularly in Europe and East Asia (5, 6). While South America was concluding its 2025 influenza season, with predominance of A(H1N1)pdm09 and localized A(H3N2) activity, the importation of Northern Hemisphere variants remained a relevant concern for inter-seasonal preparedness in the region (7). The rapid expansion of subclade K in Europe has been associated with specific amino acid substitutions in HA that may affect antigenic properties; however, its emergence represents continued seasonal antigenic drift rather than a pandemic shift (5, 8).

Its early detection in Ecuador was therefore relevant not only for monitoring the geographic spread of this emerging subclade, but also because it occurred shortly before the WHO consultation on influenza vaccine composition for the 2026–2027 Northern Hemisphere season, held in February 2026. The rapid generation and public sharing of viral genomic data can complement GISRS surveillance by making regional evidence available for the broader assessment of circulating viruses that informs vaccine strain selection and subsequent preparedness activities (9).

The integration of genomic surveillance with epidemiological information is essential for interpreting travel-associated viral introductions. In this case, the molecular findings were consistent with official statements from the Ecuadorian Ministry of Public Health (MoPH), which provided the necessary epidemiological context regarding the origin of the case and informed national preparedness measures for a potential increase in influenza activity (10, 11).

## Methods

### Data acquisition

The complete hemagglutinin (HA) gene sequence of the Ecuadorian isolate was obtained from the GISAID Initiative database. The sequence analyzed corresponds to an isolate generated by the National Influenza Centre of Ecuador and subsequently made publicly available through GISAID as part of an open-data genomic surveillance framework. To construct the comparative dataset, the Ecuadorian HA sequence was used as a query in a BLAST search, and the 250 most closely related sequences based on nucleotide similarity were selected for phylogenetic analysis (*n* = 251, including the Ecuadorian isolate). This approach ensured that the dataset captured the closest evolutionary relatives of the study sequence, allowing high-resolution inference of its phylogenetic placement. Multiple sequence alignment was performed using MAFFT, and phylogenetic model selection was conducted using IQ-TREE v2.2.2 (12, 13).

The complete list and associated metadata of the 251 HA sequences included in the phylogenetic analysis are provided in Supplementary Table S1. The dataset is permanently registered in GISAID under EPI_SET_260607yq and Doi: 10.55876/gis8.260607yq

### Bayesian phylodynamic inference

A Bayesian phylogenetic analysis was performed using BEAST v1.10.4 (14). The XML input file was generated in BEAUti (15). The model was run as a single MCMC analysis with a chain length of 100 million steps, including 251 viral sequences. A Bayesian Skyline coalescent tree prior was applied, under a strict molecular clock model with a GTR substitution scheme. The prior distribution for the clock rate was set as a lognormal distribution, following standard practice for influenza molecular evolution. Convergence and effective sample sizes (ESS) were assessed in Tracer v1.7 (16), and trees were summarized with TreeAnnotator and visualized in FigTree v1.4.4 (17).

### Amino acid substitution screening and annotation

Complementary amino acid substitution screening and annotation was performed using FluSurver, a specialized tool available through the GISAID influenza surveillance platform (18). All eight genomic segments of the Ecuadorian isolate were screened for amino acid substitutions relative to the reference strains implemented in the tool. FluSurver was then used to retrieve available mutation-associated annotations from influenza surveillance records and published evidence when available. Substitutions considered most relevant for molecular surveillance were selected for reporting and are summarized in Table 1. These annotations may include reported associations with antigenic drift, host interaction, glycosylation changes, structural features, or antiviral susceptibility. FluSurver outputs were interpreted as surveillance-oriented information and should not be considered isolate-specific experimental confirmation of phenotype.

**Table 1:** Amino acid substitutions and FluSurver-derived annotations for sequence EPI_ISL_20293296.

Protein/segment	Mutation	Interest level	Reported relevance	Structural category	Glycosylation annotation
HA1	S160N	2	Antigenic drift / escape mutant.	^Antibody recognition sites; viral^ oligomerization	Creates a new potential N-glycosylation site at position 160.
_HA1_	_I176K_	_2_	Antigenic drift / escape mutant; associated with host specificity shift. Host specificity shift and antigenic drift / escape mutant	Antibody recognition sites; binding small ligands, Antibody recognition sites; viral. Oligomerization	^—^
HA1	Q189R	1	Structural modification.	Viral oligomerization; binding small ligands.	—
_HA1_	_Y211F_	_1_	Potential cellular immune response alteration.	T-cell epitope presented by MHC molecules.	—
HA1	A320T	1	Structural stabilization.	Viral oligomerization interfaces.	—
HA1	T344A	1	Post-translational stability.	Viral oligomerization interfaces.	—
HA1	S394N	1	Host-pathogen interaction.	Antibody recognition; binding host proteins.	—
NA	N358D	2	Clinical Alert: Linked to mild drug resistance to neuraminidase inhibitors	Binding small ligands (antivirals).	—
NA	D346G	0	Antigenic drift / escape mutant.	Viral oligomerization; binding small ligands.	—
NA	H468R	1	Structural integrity.	Viral oligomerization; binding small ligands.	—
PA	K133E	1	Replication efficiency.	Viral oligomerization; binding small ligands.	—
M2	D88A	0	Matrix protein stability.	—	—

This table summarizes amino acid substitutions identified across HA1, neuraminidase (NA), polymerase acidic protein (PA), and matrix protein M2 using FluSurver. For each substitution, the table reports the mutation, the FluSurver interest level, the available mutation-associated annotation, the annotated molecular or structural category, and the glycosylation annotation when reported by the tool. Substitutions are reported relative to the reference strains implemented in FluSurver, including A/Singapore/GP20238/2024 for HA and NA and A/Croatia/10136RV/2023 for internal proteins. FluSurver annotations were used for molecular surveillance interpretation and should not be considered direct experimental confirmation of phenotype for the Ecuadorian isolate.

## Results

### Phylogenetic analysis and epidemiological correlation

**Figure 1:**
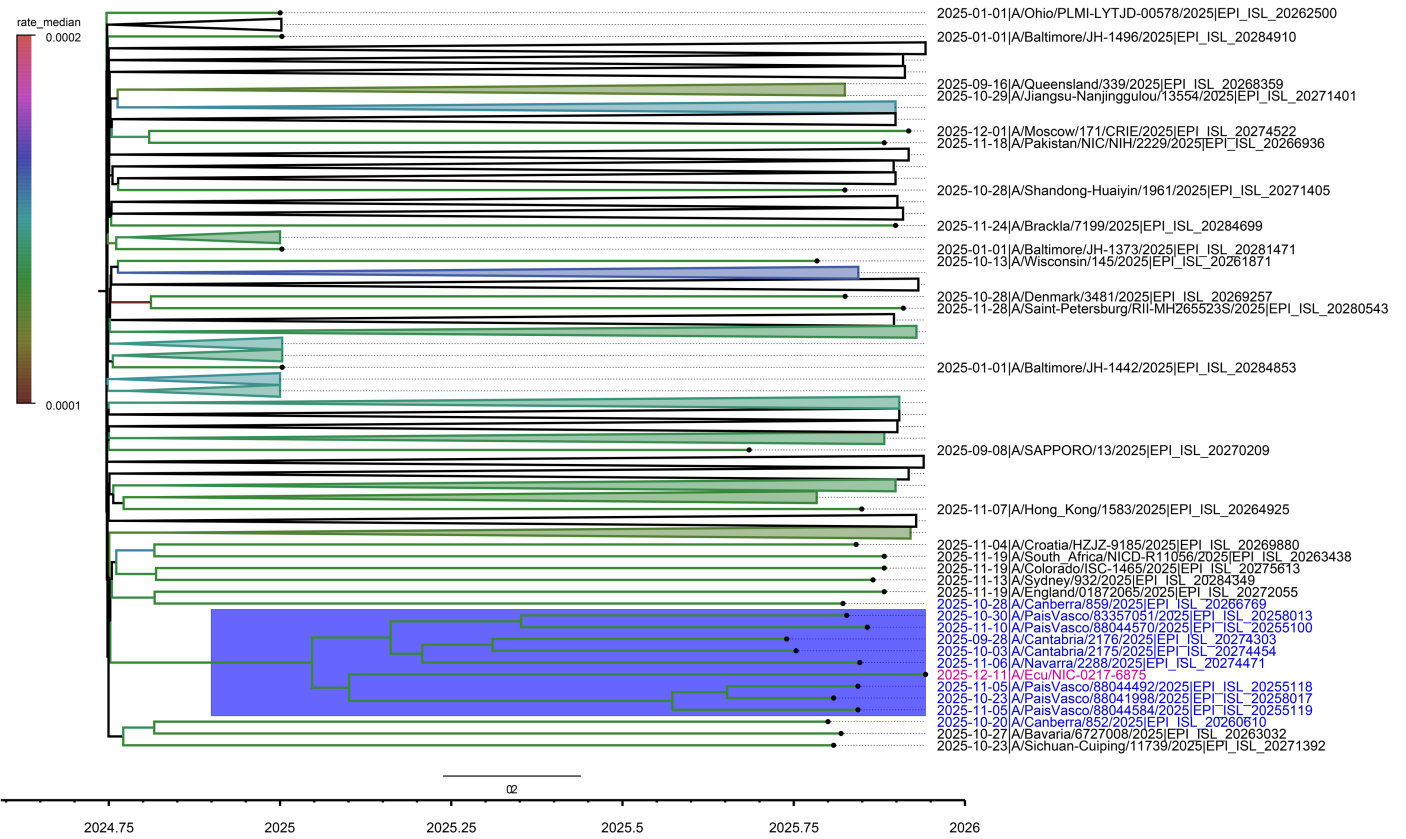
Time-scaled phylogenetic reconstruction of Influenza A(H3N2) sequences. Time-resolved phylogenetic tree of representative Influenza A(H3N2) viruses labeled with their collection date, virus designation, and corresponding accession identifiers from the GISAID Initiative database when available. Each terminal node displays the sampling date followed by the virus name and, when available, the corresponding GISAID accession identifier (format: date | virus name | accession ID). The horizontal axis represents calendar time spanning approximately from 2024.5 to 2026, whereas branch lengths reflect genetic divergence according to the scale shown in the figure (rate_median values between 0.0001 and 0.0002 substitutions per site). The dataset includes viruses collected during 2025 from multiple geographic regions, including Australia (Canberra, Sydney, Queensland), Pakistan, Japan (Sapporo), Spain (Cantabria, Navarra, País Vasco), the United Kingdom (England, Brackla), Germany (Bavaria), Denmark, Croatia, China (Sichuan-Cuiping, Shandong-Huaiyin, Jiangsu-Nanjinggulou, Hong Kong), Russia (Saint Petersburg, Moscow), South Africa, and the United States (Baltimore, Wisconsin, Colorado, Ohio). The sequence A/Ecu/NIC-0217-6875, collected on 11 December 2025, is highlighted in red and corresponds to the virus analyzed in this study. Sequences shown in blue represent closely related reference viruses included to provide phylogenetic context and illustrate the evolutionary relationships surrounding the Ecuadorian isolate within the reconstructed tree.

The MCC tree placed the isolate within subclade K (J.2.4.1), showing high affinity with sequences from Northern Spain (Basque Country and Navarra) (Figure 1). Consistent with our phylogenetic analysis, the sample came from a patient that travelled recently to Spain when this subclade was circulating.

### Molecular characterization

To describe the molecular profile of the Ecuadorian isolate, amino acid substitutions were screened and annotated using FluSurver. In the HA1 subunit, the isolate presented multiple mutations (S160N, N174D, I176K, Q189R, Y211F, A320T, T344A, and S394N). In the neuraminidase (NA) protein, the N358D, D346G and H468R substitutions were found.

In addition, internal protein analysis revealed the K133E substitution in the polymerase acidic (PA) subunit, as well as the D88A change in the matrix protein M2.

All these mutations, together with their associated annotations based on available data from the FluSurver tool, are summarized in Table 1, ensuring transparency and traceability of the genomic characterization.

### Temporal dynamics

Molecular clock analysis estimated the time of introduction to be December 2025. The 95% HPD interval ranged from mid-October to mid-December 2025, but the upper bound is the most biologically plausible timeframe, as it coincides with European circulation and the patient’s documented travel history.

## Discussion

This study reports the first identified introduction of Influenza A(H3N2) subclade K into Ecuador, presented as a case study for genomic sentinel surveillance within travel medicine in support of preparedness for potential outbreaks and epidemics. The emergence of subclade K illustrates the virus’s continuous evolutionary dynamics rather than signaling a novel pandemic threat. This finding is consistent with contemporaneous international reports on the geographic expansion of subclade K and with the documented epidemiological history of the Ecuadorian case (10, 19–21), supporting a travel-associated transatlantic introduction scenario shortly before sentinel detection in Ecuador.

The identification of this variant holds epidemiological and surveillance significance, as it highlights the speed of viral dissemination. Subclade K accounted for nearly half of the sequences reported in the EU/EEA between May and November 2025 (5). Its expansion coincided with an influenza season that began approximately 4 weeks earlier than usual in the WHO European Region, where 27 of 38 reporting countries experienced high or very high influenza activity and subclade K accounted for up to 90% of laboratory-confirmed influenza cases. Although available evidence did not indicate greater intrinsic disease severity, WHO reported significant pressure on health services in some countries, while ECDC warned that a larger epidemic could increase the absolute number of hospitalizations and strain healthcare capacity even if individual disease severity remained comparable with previous seasons (5, 22).

Its detection in South America shortly after its expansion in Europe emphasizes the efficiency of transatlantic air travel as a conduit for viral importation, even outside the typical influenza season for the Southern Hemisphere. Consistent with our molecular clock estimates and reinforcing the epidemiological context, the first case of Influenza A(H3N2) subclade K in Ecuador was officially confirmed by the Ministry of Public Health on 20 December 2025 in a patient from the Austro region who remained under medical supervision and national surveillance protocols (11). The introduction was epidemiologically consistent with recent exposure to relatives arriving from Spain, where the variant was already circulating (10, 23). Available regional reports did not indicate an unusual increase in hospitalizations or fatalities attributable specifically to subclade K, while detections in Argentina, Peru, Chile, and Bolivia supported its concurrent dispersion across South America (19, 24). In Ecuador, public health measures included strengthened respiratory-virus surveillance and expansion of influenza vaccination, with more than 716,000 individuals vaccinated by late December 2025 (25). The molecular profile of the isolate raises considerations for vaccine effectiveness. The identified HA substitutions are located in regions with reported relevance for antigenic drift or host interaction according to available mutation annotations and published evidence (5). The S160N substitution was annotated as a substitution of interest related to antigenic drift and was associated with the creation of a potential N-glycosylation site at position 160. Such glycosylation changes have been described as mechanisms that may affect antibody recognition in H3N2 viruses; however, no isolate-specific antigenic assay was performed in this study (4). N174D and I176K were annotated as substitutions with reported relevance to antigenic drift and host interaction (26). Given the reported relevance of N174D and I176K to host interaction, whole-genome amino acid substitution screening was performed using FluSurver to provide a broader molecular surveillance profile of the isolate. This analysis placed these HA substitutions in the context of additional annotated changes across viral proteins, supporting a cautious assessment of their potential public health relevance. However, FluSurver-derived annotations do not constitute isolate-specific experimental confirmation of host specificity, viral fitness, pathogenicity, or clinical severity. Reports indicate that while currently available vaccines provide protection against severe outcomes, there is antigenic distance between subclade K and the components of the 2025–2026 Northern Hemisphere vaccine (7). However, early estimates from England indicated that vaccine effectiveness against influenza-related emergency department attendance or hospitalization remained within expected ranges despite antigenic drift, reaching 72%–75% in children and adolescents and 32–39% in adults (27). Finally, while the emergence of subclade K represents expected evolutionary drift rather than a pandemic shift, its introduction into the Americas requires vigilance. Seasons dominated by A(H3N2) are historically associated with higher severity in older adults (6). Therefore, detecting this importation allows local health authorities to adjust preparedness plans, reinforcing vaccination in vulnerable groups and ensuring antiviral availability (1). Moreover, a travel medicine perspective is relevant for surveillance and control strategies in Ecuador.

In the neuraminidase (NA) protein, the N358D substitution was annotated as a mutation of interest with a reported association with neuraminidase inhibitor susceptibility. D346G and H468R were also listed in the FluSurver annotation output as substitutions with reported structural or molecular interaction categories. In addition, internal protein analysis revealed the K133E substitution in the polymerase acidic (PA) subunit, a residue implicated in replication efficiency through modulation of oligomerization interfaces and ligand-binding dynamics, as well as the D88A change in the matrix protein M2, potentially affecting ion channel stability and virion assembly (28, 29).

In conclusion, this report supports the utility of open-access genomic data combined with basic epidemiology to track pathogen movement efficiently and viral molecular evolution. The phylogenetic and epidemiological evidence supports a transatlantic introduction scenario for A(H3N2) subclade K into Ecuador. This finding served as a critical early warning for the region, demonstrating that public databases could support global health security informing in real time to public health agencies in Ecuador and South American region to enforce public health interventions during 2026 flu season.

## Data Availability

The datasets presented in this study can be found in online repositories. The names of the repository/repositories and accession number(s) can be found in the article/Supplementary material.
